# The Management of Myocardial Injury Related to SARS-CoV-2 Pneumonia

**DOI:** 10.3390/jcdd9090307

**Published:** 2022-09-16

**Authors:** Mohammed Ahmed Al-Kaif, Ahmad Naoras Bitar, Laith A. I. K. Al-Kaif, Nur Aizati Athirah Daud, Abubakar Sha’aban, Dzul Azri Mohamed Noor, Fatimatuzzahra’ Abd Aziz, Arturo Cesaro, Muhamad Ali SK Abdul Kader, Mohamed Jahangir Abdul Wahab, Chee Sin Khaw, Baharudin Ibrahim

**Affiliations:** 1 School of Pharmaceutical Sciences, Universiti Sains Malaysia, Gelugor 11800, Malaysia; mohammed.alkaif@student.usm.my (M.A.A.); dzulazri@usm.my (D.A.M.N.); faa@usm.my (F.A.A.); 2 Department of Clinical Pharmacy, Michel Sayegh College of Pharmacy, Aqaba University of Technology, South of Aqaba, South Beach Road, Opposite Aqaba Development Corporation Stores, Aqaba 910122, Jordan; ahmadnaorasbitar@live.com; 3 Department of Pharmaceutical Technology, Faculty of Pharmacy, Malaysian Allied Health Sciences Academy, Jalan SP 2, Bandar Saujana Putra, Jenjarom 42610, Malaysia; 4 Department of Medical Laboratory Techniques, Al Mustaqbal University College, Hillah 51001, Babylon, Iraq; laith1992.alkaif@gmail.com; 5 School of Medicine, Cardiff University, Heath Park, Cardiff CF14 4YS, UK; shaabana@cardiff.ac.uk; 6 Department of Translational Medical Sciences, University of Campania “Luigi Vanvitelli”, 80131 Naples, Italy; arturo.cesaro@unicampania.it; 7 Department of Cardiology, Penang General Hospital, George Town 10990, Malaysia; mdali_sheikh@hotmail.com (M.A.S.A.K.); mohamedjahangir73@yahoo.com (M.J.A.W.); khawcs@hotmail.com (C.S.K.); 8 Faculty of Pharmacy, University of Malaya, Kuala Lumpur 50603, Malaysia

**Keywords:** myocardial injury, SARS-CoV-2, management, pathogenesis

## Abstract

The global evolution of the SARS-CoV-2 virus is known to all. The diagnosis of SARS-CoV-2 pneumonia is expected to worsen, and mortality will be higher when combined with myocardial injury (MI). The combination of novel coronavirus infections in patients with MI can cause confusion in diagnosis and assessment, with each condition exacerbating the other, and increasing the complexity and difficulty of treatment. It would be a formidable challenge for clinical practice to deal with this situation. Therefore, this review aims to gather literature on the progress in managing MI related to SARS-CoV-2 pneumonia. This article reviews the definition, pathogenesis, clinical evaluation, management, and treatment plan for MI related to SARS-CoV-2 pneumonia based on the most recent literature, diagnosis, and treatment trial reports. Many studies have shown that early diagnosis and implementation of targeted treatment measures according to the different stages of disease can reduce the mortality rate among patients with MI related to SARS-CoV-2 pneumonia. The reviewed studies show that multiple strategies have been adopted for the management of MI related to COVID-19. Clinicians should closely monitor SARS-CoV-2 pneumonia patients with MI, as their condition can rapidly deteriorate and progress to heart failure, acute myocardial infarction, and/or cardiogenic shock. In addition, appropriate measures need to be implemented in the diagnosis and treatment to provide reasonable care to the patient.

## 1. Introduction

SARS-CoV-2 pneumonia has spread worldwide [1]. On 30 January 2020, the World Health Organization (WHO) declared SARS-CoV-2 pneumonia an international public health emergency [2]. Patients with chronic diseases (e.g., hypertension, coronary heart disease, and diabetes) have a high incidence of coronavirus disease 2019 (COVID-19), an increased risk of developing severe conditions, and a high mortality rate [3]. SARS-CoV-2 can also affect multiple organs throughout the body—especially the respiratory system—causing damage to some organs [4,5,6,7,8]. Acute myocardial injury has been demonstrated in 7.2–12% of patients with COVID-19 in preliminary reports [1,9]. This complication is more common among critically ill patients with COVID-19 infection, reaching 22.2–31%. The onset is often malignant, and it deteriorates rapidly. It can progress to severe heart failure, heart attack, or fatal arrhythmias in a short period, eventually leading to death [10,11,12]. Recent studies have reported that patients infected with COVID-19 might have different cardiac manifestations, such as heart muscle damage, arrhythmias, or heart attack [13]. Therefore, early identification and implementation of targeted treatment measures according to the different stages of the disease are essential to reduce the mortality of COVID-19 patients [14,15]. The SARS-CoV-2 virus is currently being studied in greater depth. This article reviews the definition, pathogenesis, and management of clinical evaluation and treatment plans to help clinicians understand the potential risk of pneumonia in terms of cardiac injury, and to promote the maintenance and management of cardiac function in MI related to SARS-CoV-2 pneumonia based on the most recent literature.

## 2. Definition of MI Related to SARS-CoV-2 Pneumonia

MI is often associated with SARS-CoV-2 pneumonia, caused by COVID-19 infection. However, the histological and pathophysiological effects of COVID-19 on the heart muscle remain unclear. MI, whether acute or chronic, is defined as an increase and/or decrease in MI markers based on cardiac troponin (cTn) concentrations >99th percentile upper reference limit (URL) [16]. Patients with dynamic changes (deltas) have an acute injury, while those without changes have a chronic injury. Delta is measured more accurately using high-sensitivity (hs) cTn assays [16].

Studies have shown that several risk factors could increase the risk of MI and mortality among COVID-19 patients. COVID-19 patients with a history of cardiovascular disease, atherosclerotic cardiovascular disease, hypertension, obesity, and/or diabetes are at a significantly higher risk of MI [17,18,19]. Furthermore, Ferrante et al., in a cohort study assessing COVID-19 patients’ chest computed tomography results, found that an increased pulmonary artery (PA) diameter was an independent risk factor for MI, and that acute MI raised the risk of mortality by twofold [20]. Additionally, acute MI among COVID-19 patients manifested in a significant increase in high-sensitivity cardiac troponin I levels [21,22]. Moreover, elevated levels of D-dimer, creatine kinase–myocardial band, and C-reactive protein were substantial predictors of MI, and were associated with a higher risk of mortality [22].

Several previous studies have determined the frequency of MI in patients with COVID-19 infection [22,23,24,25,26,27,28]. However, in the manner that many cTn studies define MI and cTn assay, the thresholds and sampling periods are often not well-defined. Furthermore, the frequency and amplitude of MI caused by SARS-CoV-2 may be underestimated, because many studies did not use hs-cTn assays and only used early single-timepoint data. It is also worth noting that several studies have employed non-directive MI definitions based on ECG or echocardiographic anomalies [1,10].

In patients with MI associated with SARS-CoV-2 pneumonia, specific problems should be analyzed in order to clarify the exact cause of the MI and adopt targeted therapy.

## 3. Mechanism of MI Related to SARS-CoV-2 Pneumonia

**Figure 1:**
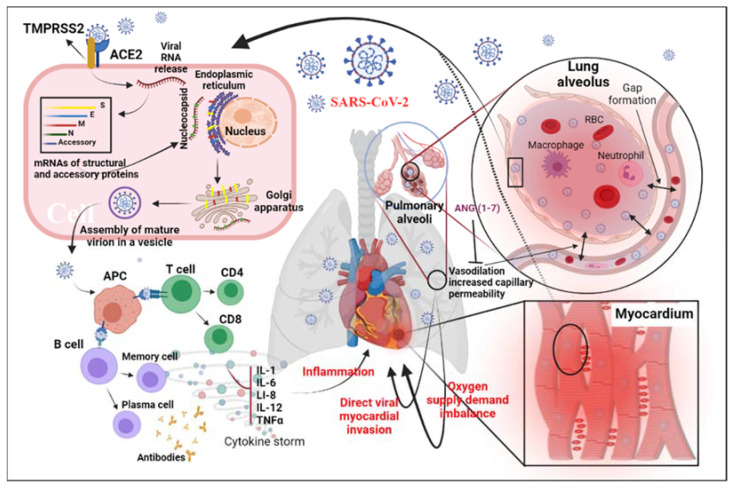
Diagram of potential physiological mechanisms of MI related to SARS-CoV-2: This figure shows the proposed mechanisms of MI related to SARS-CoV-2 infection through direct viral infection, inflammatory factors, and/or imbalance of the oxygen supply caused by acute respiratory distress syndrome. Abbreviations: ACE2, angiotensin-converting enzyme 2; S, spike; E, envelope; M, matrix/membrane, N, nucleocapsid; ANG, angiotensin; APC, antigen-presenting cell; IL-1, interleukin 1; IL-6, interleukin 6; IL-12, interleukin 12; TNF-α, tumor necrosis factor alpha; TMPRSS2, transmembrane protease, serine 2.

In patients with COVID-19 infection, the heart requires special attention. The results of histopathology in COVID-19 patients showed that the infiltrating virus particles were visible in the myocardial tissue. Still, there was no substantial myocardial damage, suggesting that the virus may not directly injure the myocardium [29]. The pathophysiological mechanism of MI associated with SARS-CoV-2 pneumonia is still controversial (Figure 1). The pathogenesis of MI may include damage to the heart muscle through direct and/or indirect action. The direct damage occurs when the virus infects the heart muscle cells by recognizing the ACE2 receptor, while the immune response may cause indirect damage. The critical component of the immune response to SARS-CoV-2 infection in macrophages is the overproduction of inflammatory cytokines such as tumor necrosis factor alpha (TNF-α) [30]. When COVID-19 infects a cell, ACE2 on the cell surface is internalized, reducing the receptor density. [31]. The reduction in surface ACE2 causes an accumulation of Ang II [32]. Furthermore, the overactivation of ADAM-17 due to the increase in Ang II binding to the Ang II type I receptors reduces the Ang II clearance, increasing the Ang II-mediated inflammatory response [31,32]. Since ACE2 receptors are involved in the pathophysiology of COVID-19, the use of renin–angiotensin–aldosterone system inhibitors for protection against COVID-19-related cardiovascular symptoms remains controversial and debated.

Whole-genome sequence analysis results show that the spike (S) protein encoded by SARS-CoV-2 contains similar ACE2 receptor-binding domains (S1) [33,34]. Audit et al., studying heart samples from coronavirus-infected mice and SARS-CoV-2 patients who died, found that SARS-CoV-2 infection in the lungs of mice could lead to ACE2-dependent MI [35]. At the same time, they found that SARS-CoV-2 RNA could be detected in 35% of heart samples of patients who died of SARS [35]. The research results from several teams also showed that SARS-CoV-2 infection, expression, and transcription are associated with ACE2 [36,37]. The aforementioned studies indicate that SARS-CoV-2 can damage myocardial cells by infecting the ACE2 receptors.

Studies have shown that cellular inflammatory factors result from an imbalance of TH1 and TH2 cytokine interactions in SARS-CoV-2 patients, and that levels of the inflammatory factors IL-4, IL-10, and IL-6 in tissue samples are elevated [1,38]. The high levels of serum cytokines—including IL-6, IL-1, IL-8, IL-12, and TNF-α—are reported to be associated with severe acute respiratory syndrome (SARS) in coronavirus infection [26,39,40,41]. Recently, research has shown that excessive T-cell activation in the peripheral blood of SARS-CoV-2 patients causes increased TH17 and high toxicity of CD8 T cells [29]. The aforementioned studies indicate that SARS-CoV-2 patients suffer from severely exaggerated immune responses.

Inflammatory factors may be involved in the process of heart failure [42]. It has been suggested that other patterns of heart muscle damage differ from those caused by direct infection with the virus. Similar reports on patients infected with the SARS-CoV-2 virus show that SARS-CoV-2 patients suffer from reversible diastolic damage related to inflammatory factors [43]. Therefore, more research on anti-inflammatory cytokines may reveal the pathogenesis of SARS-CoV-2 and inhibit inflammatory factors that may reverse heart muscle damage.

The severe symptoms that can be associated with COVID-19 infection have a significant impact on the cardiac muscle. For example, hypoxemia, respiratory distress syndrome, shock, or hypotension induced by lung infection could lead to insufficient oxygen supply to the myocardium. Furthermore, among patients with cardiac impairment or with chronic cardiovascular diseases such as coronary heart disease, the impact of these symptoms is more severe due to the increased stress on the heart and the lack of capacity to meet the required demand [44]. In addition, previous studies show that up to 20% of SARS-CoV-2 patients have an abnormal clotting function caused by MI [1,38,45]. However, the causal relationship between thrombosis and MI needs further clinical observation and clarification of pathological findings.

Elevated troponin is one of the clinical manifestations of acute MI. However, it is necessary to pay attention to its compatibility with the clinical phenotype, since troponin elevation is affected by various disease conditions [46]. Previous reports have shown that increased troponin levels may also occur after mechanical stretching induced by preload or normal cardiac physiological stress [46]. The autopsy reports of COVID-19 patients also show a small amount of infiltration of inflammatory cells in the patients’ heart tissue, with no other significant damage [29]. Therefore, further evidence is needed to support the use of troponin as a marker of direct heart injury in patients with SARS-CoV-2.

## 4. Management and Treatment of MI Related to SARS-CoV-2 Pneumonia

Generally, SARS-CoV-2 infection leads to pneumonia, associated with muscle fatigue, fever, and dry cough. Although these symptoms present as the first clinical manifestations of the infection in most patients, cardiac symptoms such as palpitations, chest tightness, and chest pain have been reported among SARS-CoV-2 pneumonia patients [30].

A significant decline has been reported in myocardial injuries, attributed to patients’ fear of visiting hospitals during the pandemic [47]. Huet et al. noted a significant decline in cases of myocardial infarction and heart failure during the SARS-CoV-2 lockdown compared to the number of cases reported before the lockdown (4.8 ± 1.6 vs. 2.6 ± 1.5 patients per day, *p* = 0.0006) [48].

A growing body of evidence has indicated that cardiac injury is common among COVID-19 patients and is associated with the severity of the disease. A meta-analysis has shown that out of 1527 SARS-CoV-2 pneumonia patients, at least 8% suffered from acute cardiac injury; furthermore, they found that SARS-CoV-2 pneumonia patients with more severe symptoms have 13 times the risk of cardiac injuries as compared to asymptomatic patients [49,50,51]. Acute myocardial infarction is a life-threatening condition that is characterized by increased high-sensitivity cardiac troponin (hs-cTn) or abnormalities in the patient’s ECG (e.g., ST elevation) [52]. According to Zeng et al. and Weltz et al., confirmed coronary revascularization for a SARS-CoV-2 pneumonia patient with ST-elevation myocardial infarction (STEMI) should be considered after evaluating the risks and benefits based on the patient’s state, with the potential to explore fibrinolytic therapy rather than percutaneous coronary intervention (PCI) [53,54,55]. However, Cameli et al. reported an increased risk of disseminating intravascular coagulation (DIC) and hemorrhagic complications associated with fibrinolytic treatment [52].

The management of cardiac patients with SARS-CoV-2 pneumonia can be challenging, and such cases may need additional care because of the higher thrombus burden. In a case series from Italy, even though the authors tried to limit PCI treatment to the severe culprit lesions and delay the non-culprit lesions until the patients’ recovery from SAR-CoV-2 pneumonia, all SARS-CoV-2 pneumonia patients with acute coronary syndromes (ST-elevation myocardial infarction (STEMI), non-ST elevation myocardial infarction (NSTEMI), and Takotsubo syndrome (TTS)) underwent angiography and were eventually treated invasively [56] (Table 1). Furthermore, all subjects received dual antiplatelet therapy with ticagrelor–aspirin, except for four subjects who received clopidogrel–aspirin. cPAP ventilation was required as respiratory support for all patients except for six, who needed endotracheal intubation (ETI), following the PCI. Herein lies the importance of using ticagrelor and clopidogrel [57,58]. In a prospective study, Choudry et al. noted that SARS-CoV-2 patients had higher levels of troponin, D-dimer protein, and C-reactive protein (1221 ng/L vs. 369 ng/L, *p* = 0.0028; 1.86 mg/L vs. 0.52 mg/L, *p* = 0.0012; and 12 mg/L vs. 50 mg/L, *p* = 0.01, respectively), while their lymphocyte counts were lower (1.3 109/L vs. 1.7 109/L, *p* = 0.0002), compared to non-SARS-CoV-2 patients [59]. Moreover, significantly higher multivessel thrombogenicity and incidence of stent thrombosis were observed, and a significantly lower left ventricular ejection fraction was detected, among SARS-CoV-2 pneumonia patients. Furthermore, a higher rate of comorbidities was detected among SARS-CoV-2 pneumonia patients, including diabetes mellitus, arterial hypertension, and hyperlipidemia.

Additionally, no significant difference was observed between groups in the total dose of heparin (SARS-CoV-2 11125 U vs. non-SARS-CoV-2 10066 U, *p* = 0.15), and similar average activated clotting time (ACT) was achieved during the procedures (*p* = 0.261); however, SARS-CoV-2 pneumonia patients needed a longer hospital stay, and were more likely to be admitted to the intensive care unit (*p* = 0.004) [59]. In a study by Bangalore et al., all 18 SARS-CoV-2 patients were reported to have elevated D-dimer and ST-segment elevation. In addition, a high prevalence of non-obstructive injuries and a poor prognosis were observed [60].

Fibrinolytic agents such as alteplase and tenecteplase were used in an international study; the authors reported successful fibrinolysis in 50 (85%) patients out of 59, with a median reperfusion time of 27 minutes [61]. Of the nine failed cases, six recovered after PCI; one died before the procedure, and two after it. Nineteen procedures were performed without using the fibrinolytic agents, and the total number of performed PCI/coronary artery bypass graft (CABG)/drug-eluting stent (DES) procedures was 28, representing 36% of COVID-19 cases, which is less than in other studies. Chinese experts recommend using fibrinolytic agents in stable patients who present to the ER within less than 12 h of the onset of symptoms and do not have any contraindications for this class of medicines [53,55]. However, many questions remain unanswered about the characteristics and indicators of the suitable populations for this approach, along with the strategies adopted and the long-term impacts or results.

Finally, Akşit E. suggested the use of ticagrelor for a patient with myocardial infarction during the pandemic for three reasons: (1) because of its pleiotropic effects, there is a lower risk due to the decreased levels of pro-inflammatory markers and the suppressed activation of platelets via the A2A and A2B adenosine receptors, reducing the chance of DIC; (2) ticagrelor showed a potential to reduce thromboinflammatory biomarkers; and (3) recent research shows that it has antibiotic potential against Gram-positive bacteria, which might increase the chances of survival in patients with coexisting diseases [62,63,64].

The histological and pathophysiological effects of COVID-19 on the heart muscle remain unclear and controversial. A histopathological analysis showed that the virus could infiltrate the cardiac muscle by utilizing the ACE2 receptor. However, the gross examination of the hearts of 51 patients showed that aside from the expected findings (i.e., mild pericardial edema and some serosanguinous pericardial effusion) from pre-existing conditions such as coronary heart disease in 29 cases, no notable abnormalities were found [65]. Furthermore, although a small number of case reports have shown that SARS-CoV-2 pneumonia can infect the myocardium, leading to viral myocarditis, the damage in the vast majority of cases was caused by increased cardiometabolic demand because of the systemic infection and ongoing hypoxia caused by severe pneumonia or ARDS [66]. Kawakami et al. concluded that the mechanism by which the virus causes cardiac damage remains uncertain, and that the infiltration by macrophages and T cells can be seen in noninfectious deaths [67].

**Table 1:** The studies that have reported on the management of myocardial injuries among SARS-CoV-2 patients.

Author	Setting	Study Design	Sample Size, N	Female, n (%)	Age (Years) *	HTN, n (%)	DM, n (%)	CKD, n (%)	Previous Myocardial Injuries, n (%)	Medications (Doses)	Coronary Intervention (PCI/CABG/DES) (%)
**Secco et al.** [56]	Italy	Prospective case series	31	7 (22.6)	72.3 ± 9	22 (71%)	12 (38.7)	-	11 (35.4)	Aspirin (500 mg), ticagrelor (180 mg), intravenous heparin (70 UI/kg)	28/31 (93.3)
**Erol et al.** [68]	Turkey	Multicenter retrospective	991	236 (23.8)	60 ± 13	499 (50.4)	335 (33.8)	-	283 (28.7)	Fibrinolytic therapy	682/991 (68.8)
**Choudry et al.** [59]	UK	Single-center prospective	39	6 (15.4)	61.7 ± 11.0	28 (71.8)	24 (61.6)	-	9 (23.1)	Heparin (5000/1000 IU)	38/39 (97.4)
**Stefanini et al.** [69]	Italy	Single-center retrospective l	28	8 (28.6)	68 ± 11	20 (71.4)	9 (32.1)	8 (28.6)	3 (10.7)	-	17/28 (61)
**Ayad et al.** [70]	Egypt	Single-center retrospective	270	50 (18.5)	57.1 ± 12.6	10 (3.7)	-	-	5 (1.8)	Ticagrelor (180 mg), clopidogrel (75 mg)	270/270 (100)
**Gluckman et al.** [71]	USA	Retrospective multicenter cross-sectional study from 6 states	1915	633 (33%)	67 ± 13	1573 (82.1)	225 (11.7)	-	395 (20.6)	-	1915/1915 (100)
**Reinstadler et al.** [72]	Austria	Multicenter retrospective	163	44 (27%)	61	(103, 63)	32 (20)	-	21 (13)	-	163/163 (100)
**Bangalore et al.** [60]	USA	Prospective case series	18	3 (17)	63	11/17 (65)	6/17 (35%)	1/17 (6%)	3/17 (17%)	Fibrinolytic agent	5/9 (56)
**Hamadeh et al.** [61]	Lithuania, Italy, Spain, and Iraq	Multicenter retrospective	78	30 (38.5)	65	57 (73)	41 (53)	69 (88.4)	9 (11)	Fibrinolytic agents (alteplase and tenecteplase)	28/78 (35.9)
**Alaarag et al.** [73]	Egypt	Single-center retrospective	26	8 (30.8)	57.7 ± 8.75	11 (42.3)	10 (38.5)	-	4 (15.4)	Ticagrelor 90 mg, clopidogrel 75 mg, aspirin 75–100 mg, heparin (70 IU/kg)	26/26 (100)
**Scholz et al.** [74]	Germany	Multicenter prospective	387	147 (28)	64.5 ± 0.7	229 (59%)	79 (20%)	26 (7%)	47 (12%)	-	352/387 (91.0%)
**Popovic et al.** [75]	France	Single-center prospective	11	4 (36.4)	63.6 ± 17.4	5 (45.5)	2 (18.2)	-	-	Aspirin (250–500 mg) heparin (70 UI/kg IV bolus), and a P2Y12 inhibitor (clopidogrel 75 mg)	11/11 (100)

*: Mean ± SD; HTN: hypertension, DM: diabetes mellitus, CKD: chronic kidney disease, PCI: percutaneous coronary intervention, CABG: coronary artery bypass graft, DES: drug-eluting stent.

## 5. The Impact of the Pandemic on the Reporting and Management of Cardiac Events 

Three large-scale multicenter registry-based studies by Erol et al., Gluckman et al., and Reinstadler et al. have shown that the COVID-19 outbreak has caused a serious fluctuation in the reporting of cardiac events. Out of 991 patients admitted to hospitals in Turkey for myocardial injuries, 682 were treated invasively with PCI, CABG, and DES [68]. Furthermore, the authors noted that the number of admissions was halved during the pandemic, and that the time to treatment increased among STEMI and NSTEMI patients during the lockdown. These observations can be attributed to the prolonged time from the onset of symptoms to the first medical contact, due to increased patient hesitancy to call the emergency medical services (EMS) for help. However, the door-to-balloon time was not affected, and a significant decline in the number of patients who went under PCI was observed in the NSTEMI group only after the outbreak of the pandemic, which can be explained by the selection of high-risk patients for this invasive procedure during this period [68]. Moreover, the increased rates of heart failure and cardiogenic shock among recruited cases led to a significant increase in the incidence of major adverse cardiovascular events (MACEs), which was associated with the delay in treatment during the pandemic.

Similarly, in the USA, the number of hospital admissions declined by 20% at the beginning of the outbreak; despite the increased admission rates later during the pandemic, the number did not return to the baseline. Furthermore, the mortality rates among STEMI patients increased, while they decreased among NSTEMI cases [71]. The results from this large-scale study (six states) validate the previous reports of significant numbers of patients dying at home or avoiding hospitals out of fear of SARS-CoV-2 pneumonia. The observed reduction in the number of admissions for treatment suggests that many patients have died without seeking medical help [76,77].

In contrast, in Austria, the number of ischemic cardiac injuries gradually increased in the first few weeks of the lockdown, which can be attributed to the increased stress during the restrictions. However, this number declined significantly from the 9th to the 12th calendar weeks. Furthermore, door-to-balloon times were not affected, remaining the same before and after the outbreak. At the same time, there was a significant increase in the ischemic time [72], indicating that healthcare system performance during the outbreak remained stable, and that healthcare workers were efficient despite the danger and the difficulties that were imposed due to the outbreak. This kind of selfless behavior was also observed among healthcare workers even in emerging countries with limited resources, such as Yemen [78].

In addition to the direct impact of the pandemic on patients with cardiovascular diseases, the indirect impacts should not be overlooked. The COVID-19 pandemic has led to the restructuring of health services to prioritize the treatment of COVID-19; therefore, patients with a previous myocardial infarction, who constitute a vulnerable group requiring continued medical attention, have experienced a reduction in their cardiac health and an increase in anxiety levels [79].

Traditional ambulatory care was disrupted by the pandemic, and many patients delayed or deferred necessary care, including preventive care. Furthermore, cardiac rehabilitation programs were temporarily closed. These changes may have resulted in delayed waves of vulnerable patients presenting for urgent and preventable cardiovascular events [80].

The management of cardiac disease is challenging by itself, let alone if the patients suffer from COVID-19 infection. These patients require extra attention, constant monitoring, and additional care. Because of the complexity of patients’ conditions, adopting a “One size fits all” approach seems unreasonable. Instead, cases should be clinically evaluated for patients with MI related to SARS-CoV-2 pneumonia, and the possible risks and benefits should be balanced before deciding which treatment strategy should be used.

## 6. Conclusions

Clinicians should give great importance to SARS-CoV-2 pneumonia patients with MI, and should identify and monitor patients with elevated troponin and/or arrhythmias as soon as possible. Acute SARS-CoV-2 pneumonia patients with MI can rapidly deteriorate and progress to heart failure, acute myocardial infarction, and/or cardiogenic shock. Comprehensive treatment measures and effective medications should be implemented as soon as possible. Implementation of respiratory support systems may have a beneficial effect in improving clinical outcomes for these patients. Studies in the scope of this topic are limited, and more randomized controlled clinical trials are still needed to provide more clinical evidence for the support and care of patients with MI related to SARS-CoV-2 pneumonia.

## Data Availability

Not applicable.
